# Splenic laceration following routine colonoscopy: a case report

**DOI:** 10.1093/jscr/rjaf940

**Published:** 2025-11-25

**Authors:** Autumn Bertch, Calvin Motika

**Affiliations:** Department of Medicine, University of North Dakota School of Medicine and Health Sciences, 1301 N Columbia Rd, Grand Forks, ND 58203, United States; Department of Critical Care and Anesthesia, CHI St. Alexius Health, 900 E Broadway Ave, Bismarck, ND 58501, United States

**Keywords:** iatrogenic splenic injury, colonoscopy, splenic laceration, post-procedural complication

## Abstract

Splenic injury is a rare but potentially life-threatening complication of colonoscopy. We report the case of a 63-year-old woman who developed left upper quadrant and referred shoulder pain one day after a routine colonoscopy. Computed tomography of the abdomen revealed a peri-splenic hematoma, and she was transferred to a tertiary facility for further management. Repeat imaging confirmed a splenic laceration with associated hemoperitoneum. She underwent splenic artery embolization with microcoils, resulting in stabilization of her hemoglobin after transfusion. The patient was discharged on hospital Day 6 without further complications. This case underscores the importance of considering splenic injury in the differential diagnosis for post-colonoscopy abdominal or shoulder pain, particularly after other more common etiologies have been excluded. Early recognition and prompt intervention can be critical in preventing morbidity and mortality.

## Introduction

Splenic injury is a rare but potentially life-threatening complication of colonoscopy. Since the first case was reported by Wherry and Zehner in 1974, the true incidence has remained difficult to determine, with modern estimates ranging from 3.2 to 14.1 per 100 000 procedures, depending on patient population and setting [1–6]. A recent meta-analysis reported a pooled rate of 0.61 per 10 000 colonoscopies (≈6.1 per 100 000), underscoring its rarity yet clinical significance [4]. Given that > 16 million colonoscopies are performed annually in the United States [5], even a very low incidence translates to a measurable number of cases each year. Colonoscopy remains the leading cause of iatrogenic splenic injury [6]. Early recognition and prompt intervention are crucial, as reported mortality rates range from 5% to 10% [1]. Written informed consent was obtained from the patient for publication of this case report and accompanying images.

## Patient case

This is a case of a 63-year-old female with a past medical history of diaphragmatic hernia, hypertension, and current 44 pack year smoking history who presented to a rural clinic with a chief complaint of left shoulder and left upper abdominal pain 1 day after undergoing a routine colonoscopy. She described her pain as sharp and radiated to her left shoulder but was not associated with dizziness, nausea, or vomiting. She denied any recent trauma. Surgical history included a prior hysterectomy.

**Figure 1:**
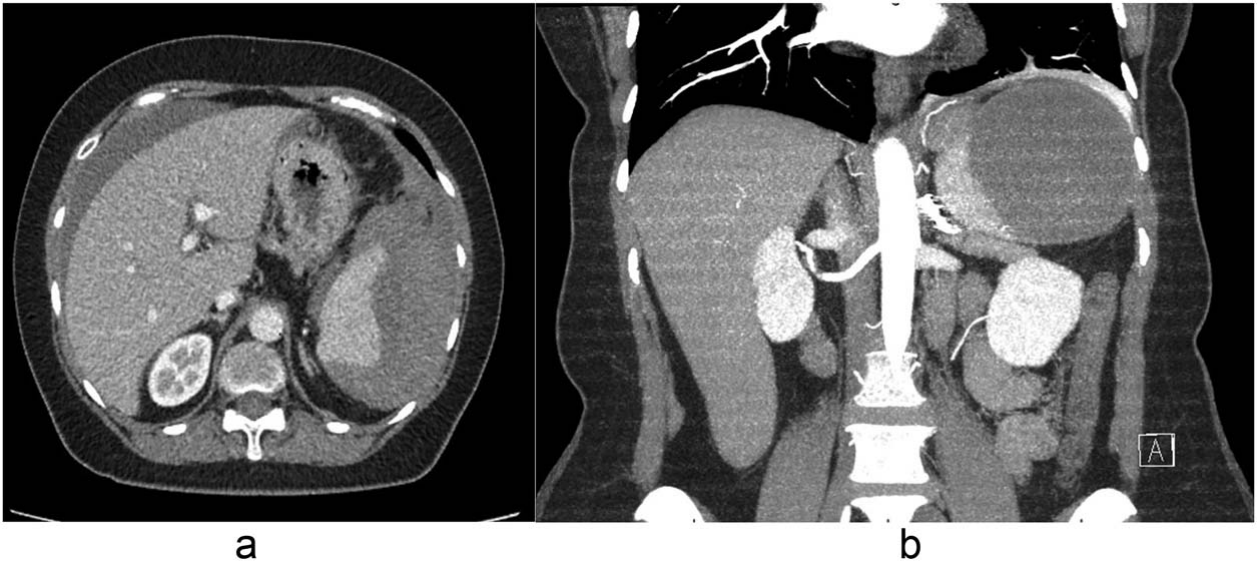
(a) Axial and (b) coronal contrast-enhanced CT images demonstrating a peri-splenic hematoma measuring 8.6 × 10.2 cm with associated hemoperitoneum.

On presentation, she was tachycardic (HR 110 bpm) with stable blood pressure. Laboratory results showed hemoglobin of 11.8 g/dl and WBC of 11.4 × 10^9^/L. Contrast-enhanced computed tomography (CT) of the abdomen demonstrated a large peri-splenic hematoma measuring 8.6 × 10.2 cm with hemoperitoneum (Fig. 1a and b). Chest X-ray and electrocardiogram were unremarkable. She was transferred to a higher-level facility, during which she developed hypotension (BP 80/40 mmHg) that responded to IV fluids.

**Figure 2:**
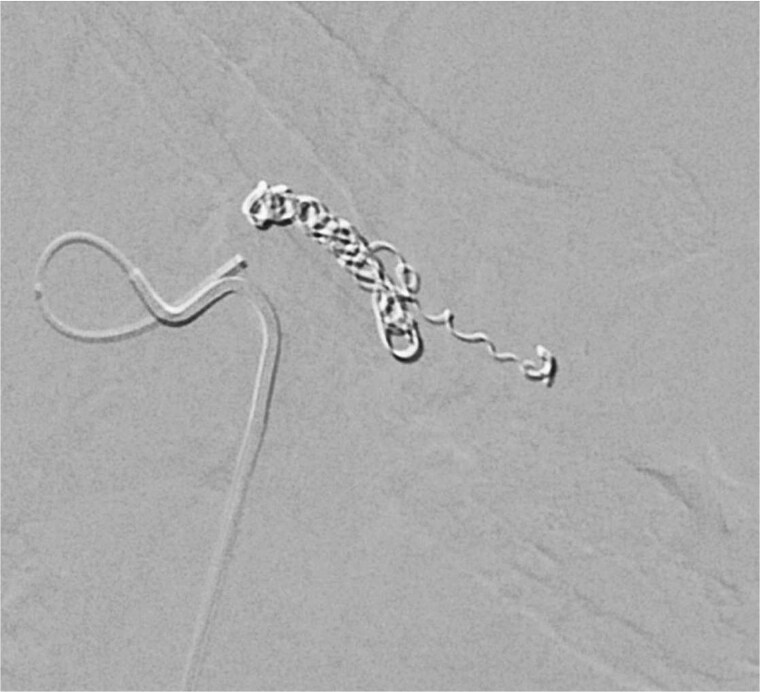
Digital subtraction angiography image demonstrating selective splenic artery microcoil embolization used to control hemorrhage while preserving collateral perfusion.

At the receiving ICU, repeat imaging demonstrated a splenic rupture with a large peri-splenic hematoma measuring 8.6 × 10.2 cm with blood tracking into the pelvis. She remained hemodynamically stable with mild left upper quadrant tenderness and no rebound or guarding. Digital subtraction angiography confirmed active splenic arterial injury, and interventional radiology performed selective splenic artery embolization with microcoils to preserve collateral flow and control hemorrhage (Fig. 2). She received two units of packed red blood cells for a hemoglobin nadir of 7.8 g/dl and was started on a 5-day course of amoxicillin–clavulanate for infection prophylaxis.

Her abdominal pain and nausea improved over the next several days. She was discharged on hospital Day 6 with instructions for outpatient follow-up, including a repeat abdominal CT in 4–6 weeks to assess for splenic infarction. Prophylactic vaccinations were recommended due to the risk of functional asplenia [7].

## Discussion

Although rare, complications of colonoscopy can be life-threatening. The most common serious gastrointestinal complications include lower gastrointestinal bleeding, post-polypectomy syndrome, and diverticulitis. Less common but significant complications include splenic hematoma or rupture, appendicitis, incarcerated hernia, subcutaneous emphysema, intramural hematoma, and ischemic colitis [1–3].

Splenic injury is thought to result from indirect trauma, particularly strain on the splenic ligaments during manipulation of the colonoscope. Capsular or ligamentous tears may occur, especially at the splenic flexure. Involvement of the splenophrenic ligament can result in referred left shoulder pain (Kehr’s sign). While historically linked to excessive force, recent evidence suggests such injuries can occur without it. Contributing factors may include looping, difficult insertion, and sedation, which can mask procedural discomfort [2].

Jehangir *et al*. reviewed 172 cases of splenic injury post-colonoscopy. The most common symptoms were abdominal and left shoulder pain, followed by dizziness, lightheadedness, and syncope. Risk factors included female sex, prior abdominal or pelvic surgery, concurrent biopsy or polypectomy, anticoagulation, and technically difficult procedures. Management included splenectomy (65.4%), embolization (6.3%), and conservative treatment (28.3%). Over 75% of patients required transfusions, with a mean hemoglobin drop >3 g/dl. Average hospital stay was 7.2 ± 5.8 days; mortality was 5.3% [1].

Patients who are functionally or anatomically asplenic require vaccination against encapsulated organisms. The Centers for Disease Control and Prevention recommends immunization with pneumococcal (PCV13 and PPSV23), meningococcal (MenACWY and MenB), and Hib vaccines [7].

## Conclusion

Splenic injury is a rare but potentially fatal complication that should be considered when other common causes of abdominal pain resulting from a colonoscopy such as gastrointestinal bleeding, post polypectomy syndrome, and diverticulitis, have been ruled out. The incidence of this complication is low, but mortality can be as high as 5%–10% [1]. Splenic injuries from colonoscopies are thought to result from indirect trauma that causes stress of the splenic ligaments resulting in tearing of the splenic capsule [1–3]. Risk factors for splenic injury resulting from colonoscopies include female sex, history of previous abdominal pelvic surgery, polypectomies performed during the colonoscopy, antiplatelet, or anticoagulant medications, or technically difficult colonoscopies [1]. The most common presenting signs are abdominal and left shoulder pain. Abdominal CT scans are considered the gold standard for evaluating suspected splenic injury. The most common management pathways for this complication include splenectomy, splenic embolization, and conservative management. If a splenectomy is performed, the patient should receive pneumococcal, meningococcal, and Hib vaccinations [7].
